# Pseudokinases, Tribbles Proteins and Cancer

**DOI:** 10.3390/cancers15143547

**Published:** 2023-07-09

**Authors:** Guillermo Velasco, Wolfgang Link

**Affiliations:** 1 Department of Biochemistry and Molecular Biology, School of Biology, Complutense University, 28040 Madrid, Spain; 2 Instituto de Investigaciones Sanitarias San Carlos (IdISSC), 28040 Madrid, Spain; 3 Instituto de Investigaciones Biomédicas “Alberto Sols” (CSIC-UAM), Arturo Duperier 4, 28029 Madrid, Spain

The human kinome comprises 518 protein kinases, of which approximately 10% lack one or more of the conserved amino acids necessary for catalytic activity. These proteins are categorized as pseudokinases and are predicted to be inactive enzymatically [1]. Pseudokinases are believed to primarily signal via noncatalytic mechanisms, such as protein–protein interactions, contributing to the development of various human diseases. Recently, this class of kinase-like proteins has emerged as a promising target for drug development [2]. Several pseudokinases have been implicated in regulating signaling pathways involving receptor tyrosine kinases, LKB1/AMPK, AKT, MAPKs, and other pathways associated with cancer initiation and progression. Among them, the Tribbles family of pseudokinases, including TRIB1, TRIB2, and TRIB3, has garnered significant attention in the past decade due to their ability to modulate inflammation, metabolism, and cancer by controlling key signaling pathways [3]. In response to this intricate landscape, the goal of this Special Issue is to consolidate recent studies and discoveries that shed light on the molecular mechanisms of cell signaling mediated by Tribbles pseudokinases in cancer, aiming to conceptualize the available evidence. This Special Issue, titled “Pseudokinases, Tribbles Proteins, and Cancer,” encompasses six original articles, seven review articles, and one commentary, each advancing knowledge in this research field.

Resistance to therapy stands as a leading cause of cancer-related deaths and a significant obstacle to effective cancer treatment. Accumulating evidence suggests that Tribbles proteins play a crucial role in mediating drug resistance in cancer cells. Several articles in this Special Issue focus on how different members of the Tribbles protein family influence drug response in cancer patients. The review article by Mayoral-Varo et al. summarizes our evolving understanding of the implications of TRIB2, the most ancestral member of the Tribbles family, in mechanisms of drug resistance [4]. The authors discuss the potential utility of TRIB2 as a biomarker for stratifying patients who would benefit most from specific treatment approaches and propose strategies for targeting TRIB2 to overcome therapy resistance. In an original research article by Örd et al., it is revealed that TRIB3 suppresses the transcriptional activity of Activating Transcription Factor 4 (ATF4), thereby promoting resistance to the proteasome inhibitor bortezomib in hepatoma cells [5]. The authors demonstrate the colocalization of TRIB3 with ATF4 on chromatin and their binding to genomic regions containing the C/EBP–ATF motif. The disruption of TRIB3 leads to enhanced C/EBP–ATF-driven transcription, increased endoplasmic reticulum (ER) stress, and cell death upon bortezomib treatment. Another original research study, conducted by Machado et al., investigates the transcriptional profile induced by the ectopic expression of TRIB2 and identifies two naturally occurring alkaloid compounds, harmine and piperlongumine, capable of reversing the TRIB2-induced gene expression pattern [6]. Consistent with previous findings that TRIB2 acts as a repressor of FOXO3 protein, these two compounds induce the activity of the FOXO3 transcription factor and revert TRIB2-mediated drug resistance. Hernández-Quiles et al. conducted a comprehensive analysis of the interactomes of mammalian Tribbles proteins, shedding light on their molecular interactions and functional roles [7]. Their findings reveal that TRIB3, a member of the Tribbles family, plays a significant role in gene repression. This study provides important insights into the regulatory mechanisms mediated by Tribbles proteins, particularly TRIB3, in the context of gene expression control. In their study, Orea-Soufi et al. investigate the role of the pseudokinase TRIB3 in the regulation of the HER2 receptor pathway and its potential as a prognostic biomarker in luminal breast cancer [8]. Their findings demonstrate that TRIB3 acts as a negative regulator of the HER2 pathway, implicating it in the modulation of HER2-driven signaling. Moreover, the study reveals that high levels of TRIB3 expression are associated with a favorable prognosis in luminal breast cancer patients. This research provides valuable insights into the role of TRIB3 as a potential therapeutic target and prognostic indicator in luminal breast cancer. Another original study by Wang et al. investigates the role of Tribbles pseudokinase 3 (TRIB3) in the cancer stemness of endometrial cancer cells and its impact on β-catenin expression [9]. Their findings reveal that TRIB3 plays a significant role in promoting cancer stemness by regulating β-catenin expression, a key protein involved in stem cell maintenance and tumorigenesis. This study provides valuable insights into the molecular mechanisms underlying the cancer stem cell phenotype in endometrial cancer and highlights TRIB3 as a potential therapeutic target for inhibiting stemness and improving treatment outcomes. The study conducted by Shahrouzi et al. explores the genomic and functional regulation of TRIB1 and its contribution to prostate cancer pathogenesis [10]. Their findings uncover the involvement of TRIB1 in prostate cancer development and progression via genomic alterations and functional dysregulation. This study provides valuable insights into the molecular mechanisms underlying prostate cancer and highlights the potential of TRIB1 as a therapeutic target for the management of this disease. The review article authored by McMillan et al. delves into the structure and function of TRIB1 and its implications in myeloid neoplasms and other related disorders [11]. The authors unravel the intricate relationship between the structural aspects of TRIB1 and its functional role in the pathogenesis of myeloid neoplasms, shedding light on the underlying molecular mechanisms. Another review article by Ferreira et al. investigates the role of Tribbles pseudokinases in colorectal cancer. Their research highlights the involvement of Tribbles pseudokinases in colorectal cancer development, progression, and therapeutic response. This article provides valuable insights into the potential use of Tribbles pseudokinases as biomarkers and therapeutic targets for colorectal cancer treatment. In their review, Stefanovska et al. explore the regulation and contribution of Tribbles pseudokinase 3 (TRIB3) in cancer [12]. Their manuscript focuses on unraveling the molecular mechanisms of TRIB3 regulation and its impact on cancer development and progression and the role of TRIB3 as a potential therapeutic target and prognostic marker in various types of cancer. In their article, Ruiz-Cantos et al. present reflections and insights from the Tribbles Research and Innovation Network, a collaborative platform dedicated to the study of Tribbles pseudokinases [12]. The authors discuss recent advancements, emerging trends, and potential future directions in Tribbles research, highlighting the importance of interdisciplinary collaboration and knowledge exchange. Likewise, in their review article, Dobens et al. discuss the role of Tribbles pseudokinase in controlling cell growth and proliferation in the model organism Drosophila, where the Tribbles gene (*trbl*) and protein were originally identified [13]. The authors shed light on the conserved functions of Tribbles across species and offer lessons from Drosophila that can contribute to our understanding of cell growth control in various organisms, including humans.

Collectively, the articles presented in this Special Issue provide valuable insights into the complex interplay between pseudokinases, specifically the Tribbles family, and cancer. By unraveling the molecular mechanisms underlying cell signaling mediated by Tribbles pseudokinases, the researchers aim to pave the way for the development of novel therapeutic strategies that can overcome drug resistance and enhance the effectiveness of cancer treatments. Likewise, the research presented in this Special Issue may also contribute to setting the bases for the potential exploration of Tribbles proteins as potential biomarkers in cancer.

## References

[1] Boudeau J., Miranda-Saavedra D., Barton G.J., Alessi D.R. (2006). Emerging roles of pseudokinases. Trends Cell Biol..

[2] Kung J.E., Jura N. (2019). Prospects for pharmacological targeting of pseudokinases. Nat. Rev. Drug Discov..

[3] Eyers P.A., Keeshan K., Kannan N. (2017). Tribbles in the 21st Century: The Evolving Roles of Tribbles Pseudokinases in Biology and Disease. Trends Cell Biol..

[4] Mayoral-Varo V., Jiménez L., Link W. (2021). The Critical Role of TRIB2 in Cancer and Therapy Resistance. Cancers.

[5] Örd T., Örd D., Kaikkonen M.U., Örd T. (2021). Pharmacological or trib3-mediated suppression of atf4 transcriptional activity promotes hepatoma cell resistance to proteasome inhibitor bortezomib. Cancers.

[6] Machado S., Silva A., De Sousa-Coelho A.L., Duarte I., Grenho I., Santos B., Mayoral-Varo V., Megias D., Sánchez-Cabo F., Dopazo A. (2020). Harmine and Piperlongumine Revert TRIB2-Mediated Drug Resistance. Cancers.

[7] Hernández-Quiles M., Baak R., Borgman A., Den Haan S., Alcaraz P.S., Van Es R., Kiss-Toth E., Vos H., Kalkhoven E. (2021). Comprehensive profiling of mammalian tribbles interactomes implicates TRIB3 in gene repression. Cancers.

[8] Orea-Soufi A., Castillo-Lluva S., Salvador-Tormo N., Martín-Cabrera P., Recuero S., Gabicagogeascoa E., Moreno-valladares M., Mendiburu-Eliçabe M., Blanco-Gómez A., Ramos-Pittol J.M. (2021). The Pseudokinase TRIB3 Negatively Regulates the HER2 Receptor Pathway and Is a Biomarker of Good Prognosis in Luminal Breast Cancer. Cancers.

[9] Wang W.L., Hong G.C., Chien P.J., Huang Y.H., Lee H.T., Wang P.H., Lee Y.C., Chang W.W. (2020). Tribbles pseudokinase 3 contributes to cancer stemness of endometrial cancer cells by regulating β-catenin expression. Cancers.

[10] Shahrouzi P., Astobiza I., Cortazar A.R., Torrano V., Macchia A., Flores J.M., Niespolo C., Mendizabal I., Caloto R., Ercilla A. (2020). Genomic and Functional Regulation of TRIB1 Contributes to Prostate Cancer Pathogenesis. Cancers.

[11] Mcmillan H.D., Keeshan K., Dunbier A.K., Mace P.D., Velasco G., Link W., O’gorman P. (2021). Structure vs. Function of TRIB1—Myeloid Neoplasms and Beyond. Cancers.

[12] Stefanovska B., André F., Fromigué O. (2021). Tribbles Pseudokinase 3 Regulation and Contribution to Cancer. Cancers.

[13] Dobens L.L., Nauman C., Fischer Z., Yao X. (2021). Control of Cell Growth and Proliferation by the Tribbles Pseudokinase: Lessons from Drosophila. Cancers.

